# Effect of Bacteriophages against Biofilms of *Escherichia coli* on Food Processing Surfaces

**DOI:** 10.3390/microorganisms12020366

**Published:** 2024-02-10

**Authors:** Ana Brás, Márcia Braz, Inês Martinho, João Duarte, Carla Pereira, Adelaide Almeida

**Affiliations:** Department of Biology, CESAM, Campus Universitário de Santiago, University of Aveiro, 3810-193 Aveiro, Portugal; ana.bras12@hotmail.com (A.B.); marciabraz96@ua.pt (M.B.); imartinho@ua.pt (I.M.); j.macedoduarte@ua.pt (J.D.)

**Keywords:** *Escherichia coli*, bacterial biofilm, bacteriophages, surface decontamination, plastic, stainless steel, food safety

## Abstract

The bacterial adhesion to food processing surfaces is a threat to human health, as these surfaces can serve as reservoirs of pathogenic bacteria. *Escherichia coli* is an easily biofilm-forming bacterium involved in surface contamination that can lead to the cross-contamination of food. Despite the application of disinfection protocols, contamination through food processing surfaces continues to occur. Hence, new, effective, and sustainable alternative approaches are needed. Bacteriophages (or simply phages), viruses that only infect bacteria, have proven to be effective in reducing biofilms. Here, phage phT4A was applied to prevent and reduce *E. coli* biofilm on plastic and stainless steel surfaces at 25 °C. The biofilm formation capacity of phage-resistant and sensitive bacteria, after treatment, was also evaluated. The inactivation effectiveness of phage phT4A was surface-dependent, showing higher inactivation on plastic surfaces. Maximum reductions in *E. coli* biofilm of 5.5 and 4.0 log colony-forming units (CFU)/cm^2^ after 6 h of incubation on plastic and stainless steel, respectively, were observed. In the prevention assays, phage prevented biofilm formation in 3.2 log CFU/cm^2^ after 12 h. Although the emergence of phage-resistant bacteria has been observed during phage treatment, phage-resistant bacteria had a lower biofilm formation capacity compared to phage-sensitive bacteria. Overall, the results suggest that phages may have applicability as surface disinfectants against pathogenic bacteria, but further studies are needed to validate these findings using phT4A under different environmental conditions and on different materials.

## 1. Introduction

Despite advances in food hygiene techniques and pathogen surveillance, foodborne diseases are still a serious global public health problem [1,2]. The World Health Organization (WHO) estimates that around 420,000 people die each year from foodborne diseases, with an impact of USD 110 billion on the global economy [3].

*Escherichia coli* is a non-pathogenic commensal bacterium known for its adaptability and diversity due to its ability to colonize both human and non-human gastrointestinal systems [4]. However, certain *E. coli*, particularly *E. coli* O157:H7, are pathogenic and produce toxins that cause disease with severe symptoms [5]. This bacterial strain has a great capacity to develop resistance since it accumulates resistance genes, mostly through horizontal gene transfer, being frequently multidrug resistant [6]. Pathogenic *E*. *coli* strains become aggregated and are difficult to eradicate due to the formation of biofilms [7]. It is well known that bacteria in biofilms behave differently from planktonic cells, particularly in response to antibiotic treatment, being able to persist and re-emerge after drug exposure due to many factors, including reduced penetration of the chemicals in biofilms [8].

Pathogenic *E. coli* is one of the major bacterial contaminants associated with foodborne infections [1,2], causing serious harm to human health and huge losses to the food industry and economy. This bacterium is transmitted almost exclusively through fecal contamination of food and water but can also be transmitted through human contact during food processing or cross-contamination of food surfaces [9].

According to the European Food Safety Authority (EFSA), Shiga toxin-producing *E. coli* (STEC) was the third most frequently detected pathogen in food companies in the European Union in 2021, with *E. coli* O157:H7 being one of the most problematic STEC serogroups [5]. One of the major problems in controlling STEC outbreaks is the low infectious dose required to cause infection. This means that even minor contamination of surfaces or work areas can lead to serious infection, posing a significant public health risk. Of the 6084 cases of human STEC infections, 901 hospitalizations and 18 deaths have been reported, mainly due to consumption of contaminated food and water and contact with infected animals. Approximately 5 to 10% of people infected with *E. coli* O157 develop hemolytic uremic syndrome and acute renal failure. In some cases, dialysis treatment and, in more severe cases, kidney transplantation may be required, reducing the quality of life [10,11] and leading to high treatment costs [2,12].

The formation of *E. coli* biofilms has a significant impact on industrial processes, with negative consequences for food safety and subsequent economic losses [13]. The biofilm increases the possibility of cross-contamination with *E. coli* in the food [11]. Some studies have shown that *E. coli* biofilm is present in almost all phases of the processing and production of food and can cause biological contamination of piping and damage in the equipment, therefore contaminating food [7,8,9,10]. Biofilm is composed of microbial colonies and self-produced extracellular polymeric substances (EPS), where microorganisms of the same or different species are spontaneously involved in their self-produced EPS matrix, which provides resistance to the external environment stress and reduces the effectiveness of mechanical action and commonly used disinfectants [14,15,16]. Studies show that once a microbial biofilm is formed, the resistance of bacteria to disinfectants increases by 500 times, suggesting that, in the presence of biofilms, the exposure time and concentration of disinfectants must be increased by 10–100 times to kill bacteria, when compared to bacteria in its planktonic state.

STEC biofilms have been associated with an increased tolerance to common sanitizers such as chlorine and quaternary ammonium compounds, probably due to a combination of bacterial resistance mechanisms [17,18]. Several decontamination strategies, including the use of chemical disinfectants (such as sodium dichloroisocyanurate, quaternary ammonium compounds, chlorine, peracetic acid, and lactic acid), heat treatment (pasteurization), washing (water), and chilling, are commonly used during food processing to reduce the risk of pathogens entering the food chain [11,19]. However, these strategies are not completely effective. The use of chemicals can create a risk of cross-contamination, affect food quality, have a negative impact on the environment, and damage or leave residues on treated surfaces [1,12,20,21]. Moreover, the massive use of chemicals can promote the development of resistance in bacterial communities, therefore compromising food safety and, consequently, public health [22]. One of the most promising alternatives for the prevention and control of bacterial biofilm formation in the food industry is the use of phages (bacterial viruses) capable of lysing biofilm-forming bacteria.

Phages are viruses that strictly infect bacteria and are incapable of self-replication since they do not possess the metabolic machinery needed to generate energy and produce proteins, needing to take over the biochemical machinery of a permissive host cell in order to replicate [23,24]. Phages are self-replicating as well as self-limiting since they replicate exponentially as bacteria and decline when the number of bacteria decreases [25,26], being considered the most abundant biological entities on the planet. Lytic phages are used as bactericidal agents for biocontrol purposes because of their ability to infect and lyse their bacterial host cells in a highly specific manner, killing them rapidly and releasing newly formed phage particles at the end of the cycle [2,12,27,28]. This process confers phages their antibacterial activity. Phage treatment has several potential advantages over the use of disinfectants and other decontamination strategies. They can be used to lyse bacteria with the advantages of being highly specific and therefore cause less damage to the natural microbiota, safe and non-toxic/non-allergic, and have great potential in the defense and removal of pathogenic bacterial biofilms [29,30,31]. Phages are capable of destroying the bacterial hosts and, therefore, preventing the formation of biofilm [32]. Phages could also penetrate existing biofilm and eliminate the biofilm structure with or without killing the resident bacteria [32]. Some phages possess polysaccharide depolymerases, which are specific hydrolytic enzymes that can use polysaccharides or polysaccharide derivatives as substrate, facilitating the process of bacterial infection in the polymeric matrix of the biofilm by phages [12,21]. Despite the several advantages of using phages, there are still limitations regarding their application, namely the emergence of phage-resistance mechanisms by bacteria and the lack of definitive guidelines and regulations for phage application, although the latter does not affect the food industry as much as it affects other sectors.

Since 2006, when the US Food and Drug Administration (FDA) approved ListShield™, a phage-based product for the control of *Listeria* in meat and poultry products, there has been a significant increase in research and development of new phage-based technologies for the control of various pathogens in post-harvest foods [27]. This resulted in the development and approval of phage products like SalmoLyse^®^, EcoShield™, and ShigaShield™ from Intralytix Inc. (Baltimore, MD, USA); PhageGuard Listex™, PhageGuard S™, and PhageGuard E™ from Micreos Food Safety (Wageningen, The Netherlands) and SalmoPro^®^ from PhageLux (Montreal, QC, Canada) for application in food [1,33]. The application of lytic phages has also been suggested as a method of mitigating *E. coli* in foods like milk [34,35], beef [36,37,38], tomato [38], broccoli [38], spinach [38], lettuce [39,40,41], cantaloupes [41], lamb and mutton [42], and bivalves [43,44]. However, there are few studies on the use of phages to control *E. coli* biofilms on different processing surfaces [21,45,46,47,48].

Thus, this work evaluated the effect of phT4A phage on the prevention and reduction of *E. coli* biofilm formation on two different surfaces (plastic and stainless steel). Since the main concern with phage therapy is the growth of phage-resistant mutants [49], the ability of phage-sensitive and resistant bacteria to form biofilm after phage exposure was also investigated.

## 2. Materials and Methods

### 2.1. Bacterial Strain and Growth Conditions

In this work, the *E. coli* ATCC 13706 strain, the host of the phage phT4A, was used to contaminate the different tested surfaces. Another *E. coli* strain, ATCC 25922, was used to compare biofilm formation with that of the test bacterium (*E. coli* ATCC 13706), as this strain is used as a model organism for biofilm formation since it is classified in the literature as a strong biofilm producer [50,51]. Both bacterial strains were purchased from the American Type Culture Collection (ATCC). Bacterial cultures were maintained in Tryptic Soy Agar (TSA) (Liofilchem, Roseto degli Abruzzi, Italy) at 4 °C. Prior to the assays, a colony was aseptically transferred to a flask containing 30 mL of Tryptic Soy Broth (TSB) (Liofilchem, Roseto degli Abruzzi, Italy) which was then placed at 37 °C, under stirring at 120 rpm for 24 h, until it reaches about 10^9^ colony-forming units per milliliter (CFU/mL). This procedure was performed in all assays.

### 2.2. Phage Stock Preparation

The phage used in this work was phage phT4A, isolated in previous work from wastewater from the Aveiro region (station EEIS9 of SIMRIA Multi Sanitation System of Ria de Aveiro), using *E. coli* ATCC 13706 as host [52]. The new phage stock was prepared in Saline Magnesium (SM) buffer [0.1 M NaCl (Sigma-Aldrich, St. Louis, MO, USA), 8 mM MgSO_4_ (Sigma-Aldrich), 20 mM Tris-HCl (Sigma-Aldrich), pH 7.5], according to the method described by Costa et al. (2019) [53], with some modifications. Briefly, 200 µL of an *E. coli* culture in the exponential growth phase was added to 5 mL of 0.6% TSB [30 g/L TSB (Liofilchem), 6 g/L agar (Liofilchem), 0.05 g/L CaCl_2_ (Sigma-Aldrich), 0.12 g/L MgSO_4_ (Sigma-Aldrich), pH 7.4] and poured into a plate containing TSA. After solidification, 100 µL of a previously prepared phage stock was placed in Petri dishes and incubated for at least 8 h at 37 °C. This procedure was repeated on several plates. After incubation, the surface layer of the plates was removed and transferred to a flask containing SM buffer, which was incubated for 24 h at 60 rpm. Then, the entire content was distributed into falcon tubes and centrifuged at 10,000 rpm for 10 min to remove intact bacteria or bacterial debris. The phage stocks were also subjected to a filtration process using a polyether sulfone membrane with a 0.22 µm pore size. Phage suspension was stored at 4 °C.

The titer of the new phage suspension was determined using the double-layer agar technique [54]. Successive dilutions of the phage suspension were performed in Phosphate Buffer Saline solution [PBS: 137 mmol^−1^ NaCl (Sigma-Aldrich), 2.7 mmol^−1^ KCl (Sigma-Aldrich), 8.1 mmol^−1^ Na_2_HPO_4_·2H_2_O (Sigma-Aldrich), 1.76 mmol^−1^ KH_2_PO_4_ (Sigma-Aldrich), pH 7.4]. Five hundred microliters of each dilution (in duplicate) and 200 µL of fresh bacterial culture were mixed with 5 mL of 0.6% TSB and placed on TSA plates. The plates were incubated at 37 °C for approximately 8 h, and the number of plaques was counted in the most appropriate dilution. The results were expressed in plaque-forming units (PFU)/mL.

### 2.3. Formation of Biofilms

The biofilm formation on plastic or stainless steel was performed in a 96-well plate or bars (2 cm × 1 cm), respectively, based on the methods previously described by Mukane et al. (2022), Park and Park (2021) and Sadekuzzaman et al. (2017) [55,56,57] with some modifications. Briefly, 200 µL of grown *E. coli* culture were transferred to each well of a 96-well plate, or 5 mL were transferred to sterilized tubes, depending on the assays on plastic or stainless steel, respectively. The plates or tubes (after bars submerging) were incubated without agitation for 24 h at 37 °C, the ideal growth temperature for *E. coli*. At this temperature, the formation of a mature and well-structured biofilm can be expected. In addition, incubation of biofilms at optimal temperatures could trigger rapid growth of bacteria in the biofilm. After incubation, suspended cells were removed, and the wells or bars were washed three times with PBS to remove all planktonic or non-adherent bacteria. 

### 2.4. Characterization of Bacterial Capacity of Biofilm Formation

Biofilm formation was performed in 96-well microplates as described in Section 2.3. This procedure was carried out for *E. coli* ATCC 13706 and *E. coli* ATCC 25922. Biofilm formation was evaluated by crystal violet assay according to Mangieri et al. (2021), Mukane et al. (2022), and Coffey and Anderson (2014), with some modifications [56,58,59]. Briefly, the wells were stained with 0.1% crystal violet for 15 min, washed three times with PBS, and allowed to dry overnight at room temperature. As suggested by Coffey and Anderson (2014) [59], overnight drying was found to be the best option to properly dry the samples and allow for proper observation of the biofilm formation by each bacterial strain under an optical inverted microscope (MOTIC AE31). After the 96-well plates were dry, 125 µL of 30% acetic acid were added to solubilize crystal violet for 15 min. The 125 µL of solubilized crystal violet were transferred to a new microtiter plate and the optical density (OD) was read at 540 nm using a plate reader (Thermo scientific, Multiskan FC). This procedure was repeated up to the stage of staining the wells with crystal violet in new 96-well plates to observe the differences in biofilm formation between the two strains under an inverted microscope (MOTIC AE31). The OD values allowed us to assess not only the ability to form biofilm but also the amount of biofilm produced by each bacterial strain. The biofilm formation capacity parameter was evaluated according to the method by Mukane et al. (2022) [56]. This is based on comparing the absorbance value at OD_540 nm_ of the negative control (OD_C_) with the absorbance value at OD_540 nm_ of the wells containing the bacteria (OD_B_). Thus, the ability of bacteria to form biofilm can be classified into four categories: non-former (OD_B_ ≤ OD_C_), weak former (OD_C_ < OD_B_ ≤ 2OD_C_), moderate former (2OD_C_ < OD_B_ ≤ 4OD_C_), strong former (4OD_C_ < OD_C_). The parameter to evaluate the amount of biofilm formed (BF), by Mangieri et al. (2021) [58], was also applied and is based on the equation: BF = BC/CW, where BC is the absorbance value at OD_540 nm_ of stained adherent bacteria while CW is the absorbance value at OD_540 nm_ of the control wells only stained and free of bacteria BF values are categorized according to the amount of biofilm formation as strong: BF ≥ 6, moderate: 5.99 ≥ BF ≥ 4, weak: 3.99 ≥ BF ≥ 2, and negative: BF < 2.

### 2.5. Formation and Quantification of the E. coli Biofilm on Plastic and Stainless Steel

The biofilm formation on plastic or stainless steel was performed in a 96-well plate or bars (2 cm × 1 cm), respectively, as described in Section 2.3. For biofilm quantification, the plate (containing 200 µL of new TSB medium) or bars (in new sterilized tubes containing 5 mL of new TSB medium) were placed in the sonicator (BANDELIN, SONOREX SUPER RK 102 H) for 10 min. Aliquots were then collected, dilutions were made, and seeded by the drop-plate method (two drops of 10 µL each) on plates containing TSA. After the incubation period of 24 h at 37 °C, the bacterial colonies were counted, and the results were expressed in CFU/cm^2^.

### 2.6. Efficacy of the phT4A Phage in the Reduction of E. coli Biofilm on Plastic and Stainless Steel

The biofilm reduction assays were performed in 96-well plates or bars based on the methods already described in the literature for this type of assay by Park and Park (2021) and Zhu et al. (2022), with some modifications [60,61]. Biofilm formation was performed in 96-well microplates or bars as described in Section 2.3. Phage phT4A was added to the phage control and test sample (containing both bacterial biofilm and phage) at a final concentration of 10^9^ PFU/mL to obtain a multiplicity of infection (MOI) of 10. Two controls were included: bacteria control and phage control. The bacteria control was inoculated with *E. coli* but not with phage, and the phage control was inoculated with phage phT4A but not with bacteria. As food is often handled at room temperature closer to 25 °C in the food industry, test samples and controls were incubated under the same conditions at 25 °C without agitation. After 0, 6, 12, and 24 h of incubation, the 96-well plates or bars were placed in the sonicator (BANDELIN, SONOREX SUPER RK 102 H) for 10 min, and aliquots were collected and diluted in PBS. Phage titer was determined by the double-layer agar method at 37 °C after 8 h. Bacterial concentration was determined in triplicate in solid TSA medium after 24 h at 37 °C using the drop-plate method. Bacterial colonies or phage plaques were counted at the most appropriate dilution, and the results were expressed as CFU/cm^2^ or PFU/cm^2^, respectively. Three independent experiments were performed on different dates, with three replicates in each condition.

Colonies of the test samples (BP) in plastic were picked and purified by successive sub-culturing in TSA to remove attached phage particles. The bacterial sensitivity of these colonies to phage after phage exposure was evaluated using the spot test procedure. Briefly, three hundred microliters of bacterial culture previously inoculated in TSB 0.6% agar were overlaid on solid TSA and spotted with 10 µL of the phage suspension. The plates were incubated at 37 °C for 8 h. Two resistant colonies (spot test negative) and two sensitive colonies (spot test positive) were selected for further experiments (as described in Section 2.8). 

### 2.7. Efficacy of phT4A Phage in the Prevention of E. coli Biofilm on Plastic

The ability of phT4A phage to prevent biofilm formation by *E. coli* was tested according to the protocol described by Zhu et al. (2022), with some modifications [60]. To obtain a MOI of 10, bacterial suspension at a final concentration of 10^8^ CFU/mL and phage at a final concentration of 10^9^ PFU/mL were added to the wells of a 96-well plate. Two controls were included: phage control and bacteria control. The test sample and controls were incubated at 25 °C without agitation. After incubation, the plates were placed in the sonicator (BANDELIN, SONOREX SUPER RK 102 H) for 10 min. Bacterial concentration was determined at time zero and after 12, 24, 30, 36, and 48 h of incubation as described above (see Section 2.6). Phage titer in the phage control and in the presence of the host was determined at time zero and after 12 and 48 h of incubation as described above (see Section 2.6). This assay was repeated three times on different dates, with three replicates in each condition.

The formation of biofilm was evaluated according to the crystal violet method, as described in Section 2.4. The plates were observed by inverted microscopy (MOTIC AE31).

### 2.8. Evaluation of the Biofilm Formation Ability of Resistant and Sensitive Bacteria to phT4A Phage

The biofilm formation capacity of mutant phage-resistant and sensitive bacteria was evaluated according to the method described by Filippov et al. (2011), with some alterations [62], using the viable cell counting and crystal violet staining method. First, two phage-resistant and sensitive colonies from an assay of biofilm reduction were isolated (see Section 2.6). Then, resistant and sensitive bacteria and a bacteria control (bacteria without contact with the phage phT4A) were allowed to grow as described in Section 2.1. Then, the capacity of biofilm formation of these different cultures was evaluated by crystal violet assay, as described in Section 2.4. Also, the amount of biofilm formed was quantified as described in Section 2.5 at time 0 and after 24 h. Three independent experiments were done on different dates, with three replicates in each condition.

### 2.9. Statistical Analysis

Statistical analysis was performed using the GraphPad Prism 8.4.3 program, San Diego, CA, USA. Confirmation of the normal distribution of data was carried out using the Kolmogorov–Smirnov test, and homogeneity of variances was confirmed using the Welch test. To evaluate statistically significant differences between the bacteria control, phage control, and the respective treatment groups (Section 2.5, Section 2.6 and Section 2.7), a two-way ANOVA complemented with the test Sidak (α = 0.05) was performed. For the different treatments, the significance of the differences was assessed by comparing, at a given moment, the result obtained in the treatment group with the result obtained for the corresponding control group. Differences between phage-resistant bacteria, phage-sensitive bacteria, and the control group were assessed for each sampling time using a two-way ANOVA (Section 2.8). A value of *p* < 0.05 was considered statistically significant.

## 3. Results

### 3.1. Characterization of Bacterial Capacity of Biofilm Formation 

Table 1 includes the absorbance values at 540 nm necessary for further characterization of the bacterial ability to form biofilm.

**Figure 1:**
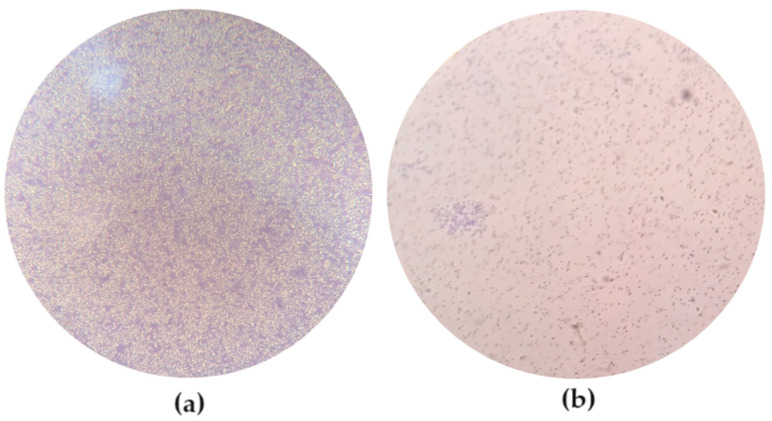
Observation of the biofilms formed by the different strains after 24 h of incubation under an inverted microscope (MOTIC AE31). (**a**) *E. coli* ATCC 13706, with 200× magnification; (**b**) *E. coli* ATCC 25922, with 100× magnification.

Considering the method that compares the OD obtained in the negative control OD_C_ to the one obtained in the wells containing the bacteria OD_B_, *E. coli* ATCC 13706 has 4OD_C_ < OD_B_ and is classified as a strong biofilm-forming bacterium. On the contrary, *E. coli* ATCC 25922 has OD_C_ < OD_B_ ≤ 2OD_C_ and is therefore classified as a weak biofilm-forming bacterium. According to the equation BF = BC/CW and the values obtained from the averaged optical density of three assays (Table 1), *E. coli* ATCC 13706 presents BF ≥ 6, and the biofilm formed by this bacterium is categorized as a strong biofilm. On the other hand, *E. coli* ATCC 25922 presents 2 ≤ BF ≤ 3.99, so the biofilm formed by this bacterium is categorized as a weak biofilm. The biofilm formed by each *E. coli* strain was observed after 24 h of incubation under an inverted microscope (MOTIC AE31) (Figure 1).

### 3.2. Efficacy of the phT4A Phage in the Reduction of E. coli Biofilm on Plastic and Stainless Steel

**Figure 2:**
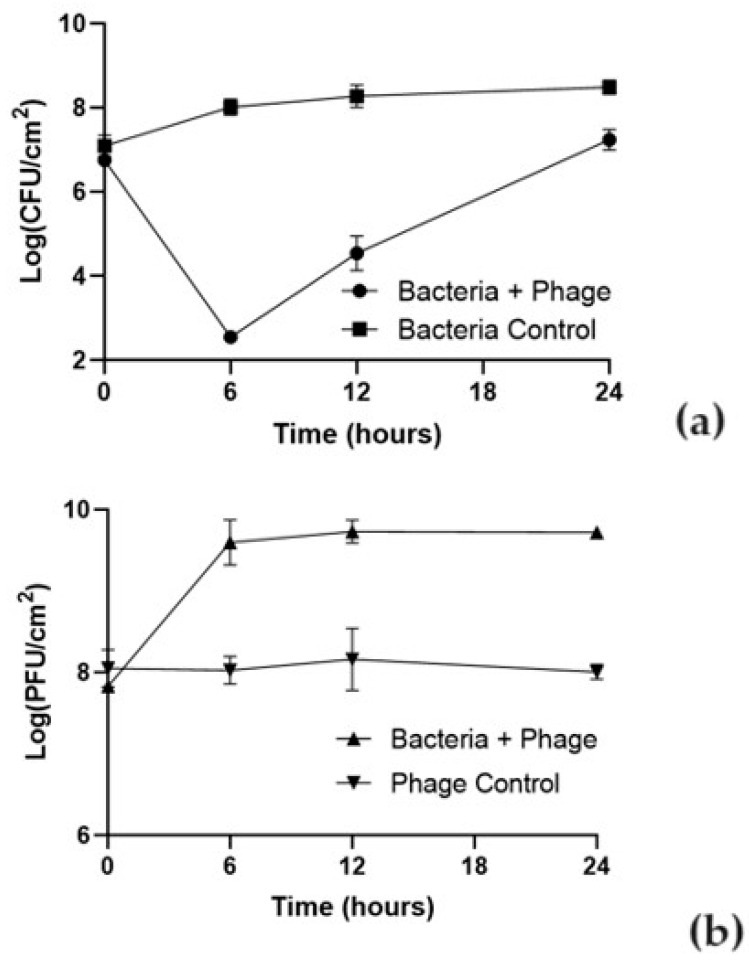
Effect of phT4A phage on the reduction of *E. coli* ATCC 13706 biofilms on plastic during 24 h at MOI 10. (**a**) bacterial concentration; (**b**) phage titer. MOI, multiplicity of infection; CFU, colony-forming units; PFU, plaque-forming units. The values shown in both graphs are the average of three independent assays, and the error bars represent the standard deviation.

**Figure 3:**
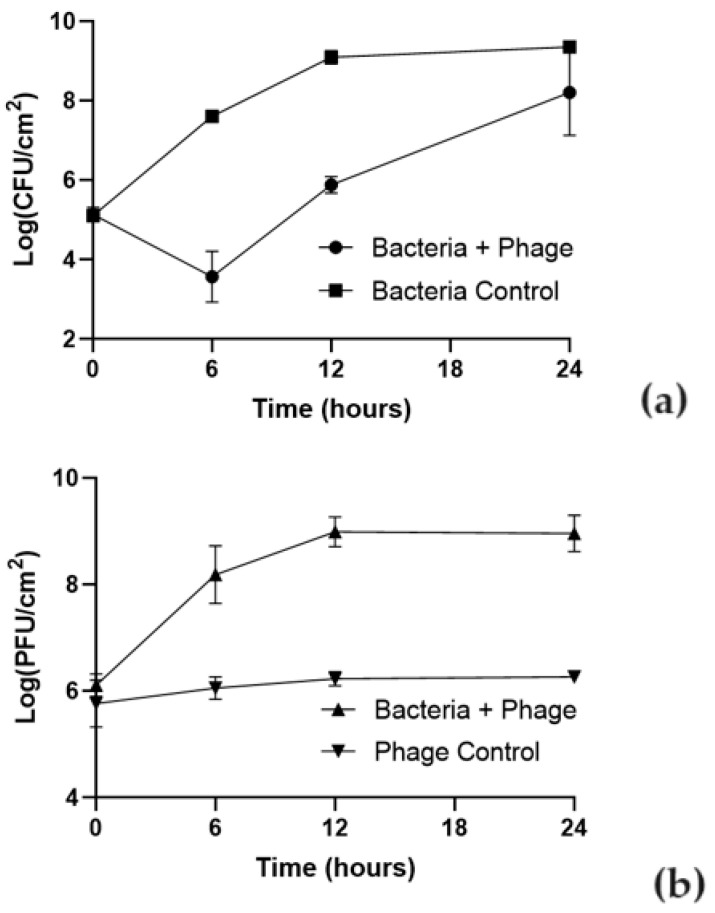
Effect of phT4A phage on the reduction of *E. coli* ATCC 13706 biofilms on stainless steel during 24 h at MOI 10. (**a**) bacterial concentration; (**b**) phage titer. MOI, multiplicity of infection; CFU, colony-forming units; PFU, plaque-forming units. The values shown in both graphs are the average of three independent assays, and the error bars represent the standard deviation.

The effect of phT4A phage on reducing *E. coli* biofilm formed on plastic and stainless steel surfaces is shown in Figure 2 and Figure 3.

The ability of the bacteria to form biofilm depended on the type of the used surface. The initial concentration of biofilm cells formed on plastic was higher (10^7^ CFU/cm^2^) than on stainless steel (10^5^ CFU/cm^2^). In the bacterial control, the biofilm cell density increased by 1.9 and 4.2 log CFU/cm^2^ in plastic and stainless steel (Figure 2a and Figure 3a), respectively, after 24 h of incubation (ANOVA, *p* < 0.05).

The *E. coli* biofilm inactivation is surface-dependent. Differences were observed in the bacterial inactivation rate between plastic and stainless steel (Figure 2a and Figure 3a). The treatment with phage phT4A significantly reduced biofilm formation by *E. coli* in 5.5 log CFU/cm^2^ (Figure 2a, ANOVA, *p* < 0.05) after 6 h of incubation. After this period, biofilm cell regrowth occurred on phage-treated plastic surfaces. Despite this bacterial regrowth, the differences in bacterial density between the test and control groups remained statistically significant (ANOVA, *p* < 0.05, Figure 2a) until the end of the experiment (decrease of 1.1 log CFU/cm^2^ after 24 h of incubation).

The maximum biofilm reduction of *E. coli* biofilm formed on stainless steel surfaces was 4.1 log CFU/cm^2^ (ANOVA, *p* < 0.05, Figure 3a) after 6 h of incubation. After this period, biofilm cell regrowth occurred on phage-treated stainless steel. Despite this bacterial regrowth, the differences in bacterial density between the test and control groups remained statistically significant (ANOVA, *p* < 0.05, Figure 3a) until the end of the experiment (decrease of 1.2 log CFU/cm^2^ after 24 h of incubation).

The initial concentration of phage adhering to plastic was higher (10^8^ PFU/cm^2^) than in stainless steel (10^6^ PFU/cm^2^) (Figure 2b and Figure 3b). The phage control (PC) remained constant throughout the experiment (ANOVA, *p* > 0.05) in plastic and stainless steel (Figure 2b and Figure 3b). In plastic, when phage phT4A was incubated in the presence of the host (Bacteria + phage), a significant increase of 1.9 log PFU/cm^2^ was observed after 6 h of incubation (ANOVA, *p* < 0.05, Figure 2b). After this period, the phage concentration remained constant until the end of the experiment. When phage phT4A was incubated in the presence of *E. coli* in stainless steel, a significant increase (ANOVA, *p* < 0.05, Figure 3b) in phage titer of 2.9 log PFU/cm^2^ was observed in the first 12 h of incubation. After this increase, the phage titer remained constant until the end of the experiment.

### 3.3. Efficacy of phT4A Phage in the Prevention of E. coli Biofilm on Plastic

**Figure 4:**
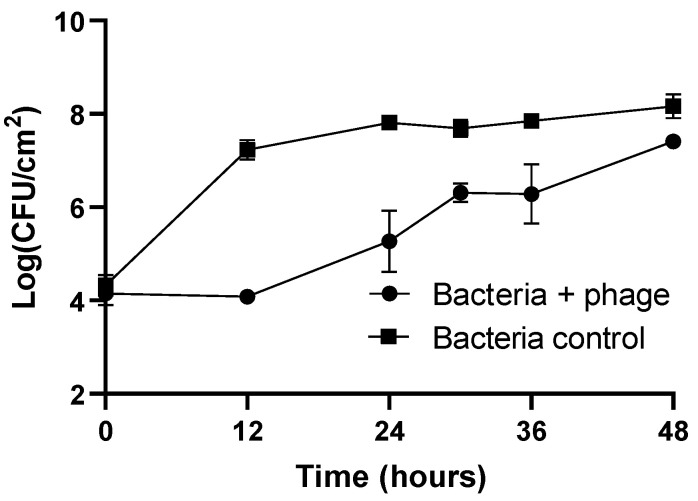
Evaluation of phage phT4A efficacy in preventing *E. coli* ATCC 13706 biofilm formation on plastic during 48 h. CFU, colony-forming units. The values shown in the graph are the average of three independent assays, and the error bars represent the standard deviation.

The results of the prevention of biofilm formation on plastic are presented in Figure 4. In the bacteria control, the biofilm cell density increases by 3.8 log CFU/cm^2^ (ANOVA, *p* < 0.05, Figure 4) during 48 h of the experiment.

The concentration of biofilm cells in the phage-treated group remained constant during the first 12 h of incubation and was similar to the initial concentration (10^4^ CFU/cm^2^). When comparing the biofilm cell density in the phage-treated group with the untreated group, a maximum reduction of 3.2 log CFU/cm^2^ was observed after 12 h of incubation (ANOVA, *p* < 0.05, Figure 4). The regrowth of biofilm cells in the phage-treated group was observed after 12 h of incubation. However, at the end of the experiment, the biofilm cell density in the phage-treated group (Bacteria + phage) and bacteria control was significantly different (reduction of 0.8 log CFU/cm^2^, ANOVA, *p* < 0.05).

The initial concentration of phage adhering to plastic was 5.1 log PFU/cm^2^. The phage control (PC) remained constant throughout the experiment (ANOVA, *p* > 0.05). When phage phT4A was incubated in the presence of the host, a significant increase of 3.3 log PFU/cm^2^ was observed after 12 h of incubation (ANOVA, *p* < 0.05). After this period, the phage concentration remained constant until the end of the experiment.

The ability of phT4A phage to prevent biofilm formation by *E. coli* was also evaluated using crystal violet staining. These results allowed us to observe the differences between the phage-treated group (Bacteria + phage) and the untreated group (Bacteria control) at time 0 and after 12, 24, 30, 36, and 48 h of treatment by observation under inverted microscopy (Table 2).

### 3.4. Evaluation of the Biofilm Formation Ability of Resistant and Sensitive Bacteria to phT4A Phage

**Figure 5:**
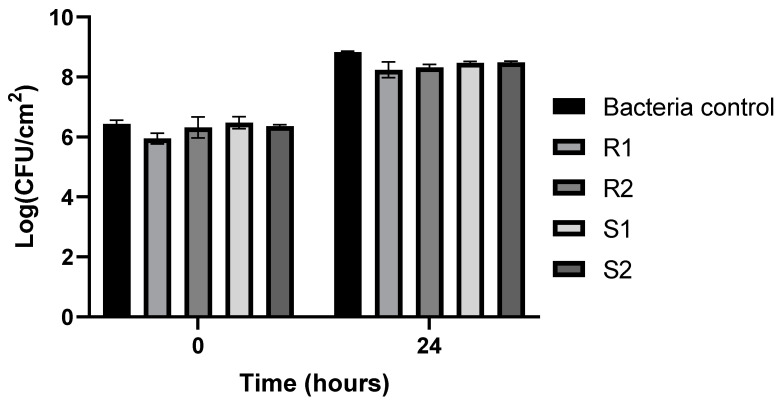
Assessment of the biofilm formation capacity of resistant and sensitive bacteria to phT4A phage during 24 h of incubation. R1 and R2, bacteria resistant to phT4A phage after contact with phage; S1 and S2, bacteria sensitive to phT4A phage after contact with phage; Bacteria control, bacteria without contact with phage phT4A; CFU, colony-forming units. The values shown in the graph are the average of three independent tests, and the error bars represent the standard deviation.

The biofilm-forming capacity of resistant and sensitive bacteria to phT4A phage is shown in Figure 5. At the end of the experiment, the biofilm density in the group of resistant bacteria (R1 and R2) was significantly lower than in the bacterial control (ANOVA *p* < 0.05, Figure 5). However, no differences (ANOVA *p* > 0.05, Figure 5) were observed between the group of resistant bacteria (R1 and R2) and the group of sensitive bacteria (S1 and S2).

**Figure 6:**
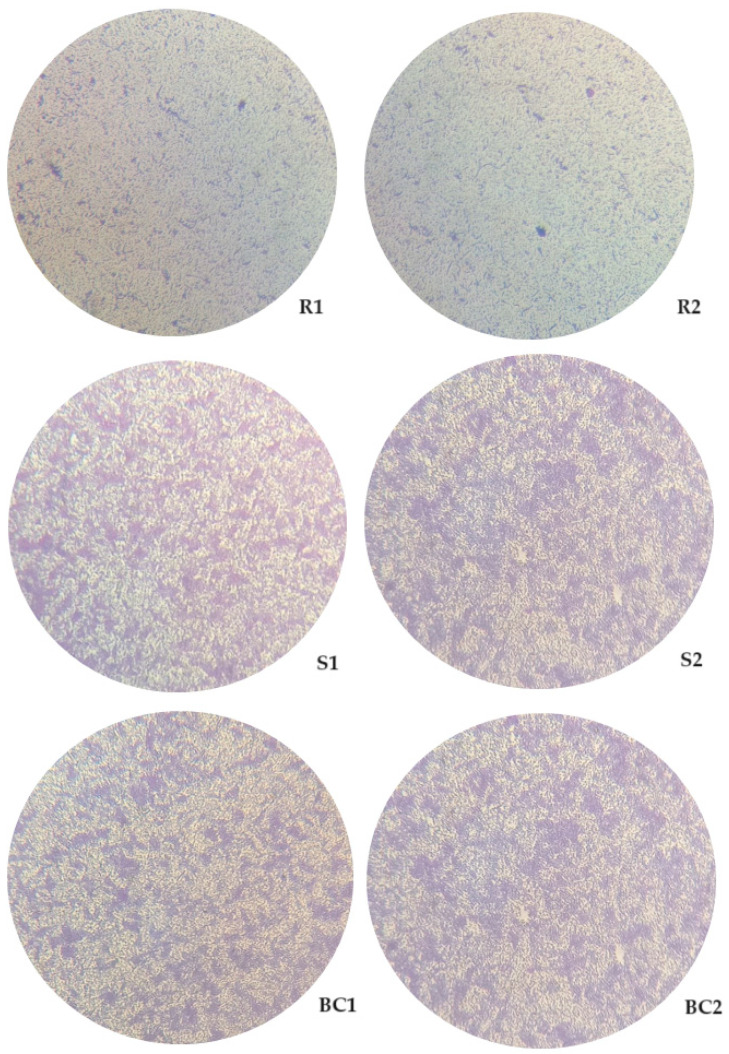
Observation of the biofilm formation on plastic by different groups of bacteria (phage-resistant, phage-sensitive, and control), with 24 h of incubation, under an inverted microscope (MOTIC AE31) with 200× magnification. R1 and R2, Bacteria resistant to phT4A phage after contact with phage; S1 and S2, Bacteria sensitive to phT4A phage after contact with phage; BC1 and BC2, Bacteria without contact with the phage phT4A.

The ability of resistant and sensitive bacteria to phage phT4A to form biofilms was observed by inverted microscopy (Figure 6). The microscopy images showed that the group of bacteria resistant to phages phT4A (R1 and R2) had a lower bacterial cell density compared to the group of bacteria sensitive to phages phT4A (S1 and S2, Figure 6). However, the groups of sensitive bacteria (S1 and S2) did not show any significant differences when compared to the control group (BC1 and BC2, Figure 6).

## 4. Discussion

The formation of biofilms on food processing surfaces by *E. coli* is a major challenge to the food industry since they are often associated with foodborne outbreaks that have a tremendous impact on health and the economy [13]. The potential use of phages to control bacterial biofilms has received increasing attention as resistance to conventional disinfectants continues to increase [63]. In the present study, phage phT4A was applied to prevent and reduce *E. coli* biofilm on plastic and stainless steel. Phage phT4A is a safe biological control agent since it does not code for integrase genes nor genes encoding for virulence factors and antibiotic resistance [52]. The results indicate that phage phT4A was effective in reducing *E. coli* biofilm on plastic and stainless steel and preventing *E. coli* biofilm formation on plastic.

The bacterial strain used in this study (*E. coli* ATCC 13706) is a strong biofilm-forming bacteria, namely when compared with the *E. coli* ATCC 25922 strain, which is considered a model of biofilm-forming bacteria in the literature [50,51]. Considering that the optimal growth temperature for *E. coli* is around 37 °C, it would be expected that this temperature would result in a more mature and well-structured biofilm. This can lead to the reduced effectiveness of phages in eradicating this mature biofilm since phages need to bind to the host bacteria to infect them. However, when phages can penetrate the biofilm formed at 37 °C, due to the supposed higher bacterial density compared to the biofilm formed at 25 °C, there is a possible higher phage replication capacity, which could result in more effective inactivation. On the other hand, incubation at 25 °C may not provide optimal growth of the bacteria and thus negatively influence the cohesion strength of the biofilm. In the context of the food industry, which is our focus, food is often handled at room temperature closer to 25 °C. For our work, the choice of incubation temperature must be as aligned as possible with the real one since the objective is to replicate as much as possible the conditions of the food industry; hence, the choice of an incubation temperature of 25 °C during the phage treatment assays of the biofilms. However, as biofilm formation on surfaces is influenced by temperature [64,65], it will be important in the future to understand the efficacy of phT4A phage to destroy/prevent biofilm formation at different temperatures. Likewise, the current results need to be further validated for other food processing conditions, including pH and other processing surfaces.

The initial adhesion of biofilm is dependent on the surface properties of the tested materials. In this study, the plastic (initial concentration of 7.1 log CFU/cm^2^) showed higher bacterial attachment compared to stainless steel (initial concentration of 5.1 log CFU/cm^2^). Similar results were observed by Rogers et al. (1994) [66] and Jaroni et al. (2023) [47]. Rogers et al. (1994) observed greater bacterial adhesion and biofilm formation on plastic surfaces than on stainless steel or glass surfaces [66]. More recently, Jaroni et al. (2023) observed higher STEC attachment to high-density polyethylene coupons (up to 5.6 log CFU/cm^2^) compared to stainless steel (up to 4.3 log CFU/cm^2^) [47]. Many factors affect the formation and adhesion strength of biofilms in abiotic surfaces, such as electrostatic charges, surface tension and hydrophobicity, coating, roughness or fissure depth, and porosity [67,68,69,70]. The plastic surfaces used in this study had a high hydrophobicity and a rougher surface than the stainless steel. In general, the most hydrophobic materials are those with the greatest biofilm formation. De-la-Pinta et al. (2019) observed that there is a greater production of biofilms on rougher and hydrophobic materials [71]. In the future, studies should include biofilm analysis by advanced microscopic methods such as TEM to study phage diffusion and attachment to biofilm and SEM to observe biofilm destruction in different abiotic surfaces.

The phT4A phage, at MOI 10, had a significant impact on the reduction of biofilm formed in the plastic surface (5.5 log CFU/cm^2^), namely in the first 6 h of treatment. Similar results were obtained by Zhu et al. (2022) [60], with a reduction of approximately 6 log CFU/well for a lower MOI of 0.1. However, this reduction occurred later, after 8 h, compared to our study (after 6 h). Additionally, the difference in the incubation temperature and the culture medium [37 °C for biofilm formation and 25 °C for phage treatment on TSB in our study vs. 37 °C for biofilm formation and phage treatment on Lysogeny Broth in Zhu et al. (2022)] [60], can significantly impact the results.

On stainless steel, our results highlight the effectiveness of phT4A phage, at a MOI of 10, in reducing the biofilm formed at 37 °C in 4.1 log CFU/cm^2^, with greater evidence 9 h after the beginning of treatment. In another study, by Wang et al. (2020), a similar protocol was carried out; however, biofilm with 24 h of maturation was formed at 24 °C instead of the 37 °C of our study [72]. The lower temperature of 24 °C was probably responsible for the lower reduction of 2.9 log CFU/bar even at a higher MOI of 100 [72].

Comparing the results obtained on both surfaces (plastic and stainless steel), it is possible to observe that at the same MOI value, the reduction of biofilm on plastic surfaces is more pronounced than on stainless steel surfaces. These differences suggest that phages possibly adhere differently to each type of surface. In this study, the plastic (initial concentration of 8.0 log PFU/cm^2^) showed higher initial viral attachment compared to stainless steel (initial concentration of 6.0 log PFU/cm^2^). However, the number of phage particles of phT4A when incubated with *E. coli* increased by 1.9 and 2.9 log PFU/cm^2^ in plastic and stainless steel surfaces, respectively. These results demonstrate that high initial phage doses may not be essential due to the self-perpetuating nature revealed by increasing phage titer. The results suggested that phT4A phage behaved differently on each surface. Probably, the biofilm matrix formed in the metal surface trapped the phages, which hinders phage penetration and adsorption (Abedon 2016). Furthermore, considering the greater bacterial adhesion in plastic, this can also explain the better inactivation results on this surface compared to the results on stainless steel. When phages can penetrate the formed biofilm, due to the supposed higher bacterial density in plastic compared to the biofilm formed on stainless steel, there is a possible higher phage replication capacity, which could result in more effective inactivation on plastic. This indicates that to translate the phage application to the routine, it is important to test the treatment on the different processing surfaces used in the industry.

In addition to biofilm reduction, the prevention of biofilm formation on food processing surfaces is also crucial to guarantee the safety and quality of food products. Our results indicated that phT4A phage prevented biofilm formation by *E. coli* ATCC 13706. Furthermore, the prevention was maintained up to 12 h of post-exposure to the phage. However, over time, a decrease in biofilm prevention capacity was observed. This may be due to the emergence of phage-resistant bacteria, limiting its effectiveness [73].

Overall, the application of phages in a coating surface can be an effective strategy to avoid biofilm formation, namely on food-handling surfaces. The presence of pores on the coating surface and the ability to control their pore size may allow the incorporation and consequent controlled release of phages from these pores to treat the coated surfaces. Also, several antibacterial additives such as silver, copper, and zinc ions can be added to the coatings and have a combined effect with phages in preventing biofilm formation on different surfaces. However, phage viability in the presence of these ions needs to be previously addressed in order to test the combined approach, preventing phage damage along with the bacteria [74].

In general, in both assays of biofilm reduction and prevention, there was a significant decrease in the bacterial concentration in the presence of phage, relative to the bacteria control, within the first hours of treatment. However, after this period, bacterial regrowth was observed. This can be attributed to (i) some bacterial cells located in the biofilm structure that can be inactive, hindering phage multiplication [72,75], and (ii) the development of phage-resistance mechanisms by the bacteria, allowing them to survive and multiply again [75]. The application of a phage cocktail containing several active phages for the same bacterial strain can prevent the development of bacterial resistance to phages [58,76]. The use of a cocktail of phages with the ability to bind to different bacterial receptors can help control the emergence of bacterial mutants by exerting selective pressure on bacterial populations in the biofilm and increasing the effectiveness of phage treatment. The combination of different antibacterial approaches should also be considered to prevent and combat the emergence of bacterial resistance to phages. The application of several phage doses for sanitization purposes may increase the effectiveness of the disinfection. Moreover, as phages are naturally present in the environment and coevolve with bacteria if bacteria are resistant to a particular phage, it is possible to isolate new phages to which these bacteria are sensitive [77].

Our results revealed a significant difference in the biofilm formation capacity between phage-resistant and sensitive bacteria after phage exposure. This suggests that although phage-resistant bacteria emerge during treatment, the phage-resistant bacteria showed slower growth and, consequently, a reduced ability to form biofilm. On the contrary, sensitive bacteria maintained a biofilm formation capacity similar to that of the control group. These results are in agreement with some other reports that similarly showed reductions in the growth of the phage-resistant mutants [78,79,80,81]. Bacterial resistance to phages can evolve via several different mechanisms, which vary in their specificity. Many of these resistance mechanisms impose a significant fitness cost [82,83], but these costs can vary across environments and the degree of competition for resources [84,85]. The results show that phage resistance can significantly alter bacterial growth and that phage-mediated selection is likely to be an important component of bacterial pathogenicity in nature.

Chemical disinfectants commonly used to disinfect surfaces achieve bacterial reductions of 4–5 log [20]. Considering the results obtained in this work, the phT4A phage seems to be a promising alternative to disinfectants. The use of chemical disinfectants can create a risk of cross-contamination, affect food quality, have a negative impact on the environment, and damage or leave residues on treated surfaces [1,6,12,13]. Moreover, the massive use of disinfectants can promote the development of resistance in bacterial communities, therefore compromising food safety and, consequently, public health [14]. Phages have been proposed as a class of bio-sanitizers due to several favorable attributes, including the fact that they only infect bacteria and can remain viable for long periods, which can prevent bacterial recontamination [86]. Phages have low toxicity, are environmentally friendly, are not corrosive, and do not affect the food properties (have any harmful or unpleasant odors) [20,86]. Phages are considered safe for human consumption with no associated safety issues related to oral ingestion [87]. Allergic reactions to phage administration are also rare [31]. By reducing the use of aggressive chemicals, the use of phages also reduces the impact associated with the disposal of these products, contributing to more sustainable and safe solutions.

## 5. Conclusions and Future Perspectives

Phage phT4A was effective in reducing *E. coli* biofilm on plastic and stainless steel and preventing *E. coli* biofilm formation on plastic. These results suggest that phages may have applicability as surface disinfectants against pathogenic bacteria. Further studies are needed to validate these findings using phT4A under different environmental conditions and in different materials. Phage phT4A did not prevent the occurrence of bacterial regrowth in the two tested surfaces. The results showed that phage resistance can significantly alter bacterial growth; however, these mutants were not as fit as their counterparts. The use of phage cocktails and the combination of different antibacterial approaches may help overcome this problem of bacterial resistance to phages. Our results pave the way for a new area of interest and study in the search for more sustainable and safer solutions based on phages. Reducing the use of aggressive chemicals will increase the quality and safety of food and decrease environmental impact and, consequently, the risks to public health.

In the future, it would be important to evaluate the impact of different phage concentrations and exposure times in the development of bacterial resistance to phages. Furthermore, it would also be important to explore the use of phage cocktails or even the combination of phages with disinfectants already used in routine surface disinfection. Also, in future studies, a more comprehensive exploration of different biofilm formation temperatures and culture media relevant to food processing environments could offer a better understanding of phage potential in various practical scenarios in the food industry. Assessing the broader ecological consequences of long-term phage persistence in the environment is also crucial for ensuring the sustainability and safety of phage-based interventions in food processing settings.

## Figures and Tables

**Table 1:** Optical density (OD_540 nm_) to characterize the intensity of biofilm formed.

	ATCC 13706	ATCC 25922
Stained adherent bacteria (BC or OD_B_)	1.381	0.221
Acetic acid + Crystal violet (CW)	0.087	
TSB (OD_c_)	0.040	

BC—absorbance value at OD_540 nm_ of stained adherent bacteria.

**Table 2:** Observation of biofilm formation on plastic at different incubation times under an inverted optical microscope (MOTIC AE31) with 200× magnification.

Time (h)	Bacteria + Phage	Bacteria Control
0	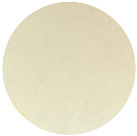	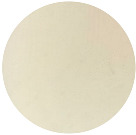
12	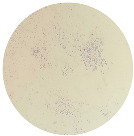	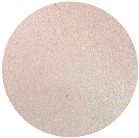
24	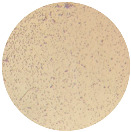	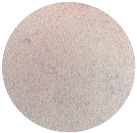
30	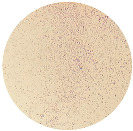	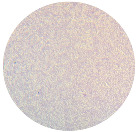
36	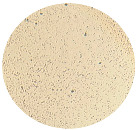	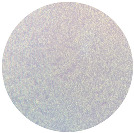
48	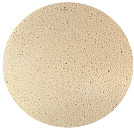	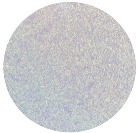

## Data Availability

Data are contained within the article.

## References

[1] Moye Z.D., Woolston J., Sulakvelidze A. (2018). Bacteriophage applications for food production and processing. Viruses.

[2] Garvey M. (2022). Bacteriophages and food production: Biocontrol and bio-preservation options for food safety. Antibiotics.

[3] WHO (2015). WHO Estimates of the Global Burden of Foodborne Diseases: Foodborne Diseases Burden Epidemiology Reference Group 2007–2015.

[4] Ramos S., Silva V., de Lurdes Enes Dapkevicius M., Caniça M., Tejedor-Junco M.T., Igrejas G., Poeta P. (2020). *Escherichia coli* as commensal and pathogenic bacteria among food-producing animals: Health implications of extended spectrum β-lactamase (ESBL) production. Animals.

[5] EFSA (2023). The European Union one health 2022 zoonoses report. EFSA J..

[6] Poirel L., Madec J.-Y., Lupo A., Schink A.-K., Kieffer N., Nordmann P., Schwarz S. (2018). Antimicrobial resistance in *Escherichia coli*. Microbiol. Spectr..

[7] Danese P.N., Pratt L.A., Kolter R. (2000). Exopolysaccharide production is required for development of *Escherichia coli* K-12 biofilm architecture. J. Bacteriol..

[8] Davey M.E., O’toole G.A. (2000). Microbial biofilms: From ecology to molecular genetics. Microbiol. Mol. Biol. Rev..

[9] Abebe E., Gugsa G., Ahmed M. (2020). Review on major food-borne zoonotic bacterial pathogens. J. Trop. Med..

[10] Yang S.C., Lin C.H., Aljuffali I.A., Fang J.Y. (2017). Current pathogenic *Escherichia coli* foodborne outbreak cases and therapy development. Arch. Microbiol..

[11] Zhou F., Wang D., Hu J., Zhang Y., Tan B.K., Lin S. (2022). Control measurements of *Escherichia coli* biofilm: A review. Foods.

[12] Gutiérrez D., Rodríguez-Rubio L., Martínez B., Rodríguez A., García P. (2016). Bacteriophages as weapons against bacterial biofilms in the food industry. Front. Microbiol..

[13] Sharan M., Vijay D., Dhaka P., Bedi J.S., Gill J.P.S. (2022). Biofilms as a microbial hazard in the food industry: A scoping review. J. Appl. Microbiol..

[14] Flemming H.C., Neu T.R., Wozniak D.J. (2007). The EPS matrix: The “house of biofilm cells”. J. Bacteriol..

[15] Branda S.S., Vik Å., Friedman L., Kolter R. (2005). Biofilms: The matrix revisited. Trends Microbiol..

[16] Carpio A., Cebrián E., Vidal P. (2019). Biofilms as poroelastic materials. Int. J. Non Linear Mech..

[17] Wang R., Bono J.L., Kalchayanand N., Shackelford S., Harhay D.M. (2012). Biofilm formation by Shiga toxin–producing *Escherichia coli* O157:H7 and non-O157 strains and their tolerance to sanitizers commonly used in the food processing environment. J. Food Prot..

[18] Fouladkhah A., Geornaras I., Sofos J.N. (2013). Biofilm formation of O157 and non-O157 Shiga toxin-producing *Escherichia coli* and multidrug-resistant and susceptible *Salmonella* Typhimurium and newport and their inactivation by sanitizers. J. Food Sci..

[19] Bumunang E.W., Zaheer R., Niu D., Narvaez-Bravo C., Alexander T., McAllister T.A., Stanford K. (2023). Bacteriophages for the targeted control of foodborne pathogens. Foods.

[20] Vikram A., Woolston J., Sulakvelidze A. (2020). Phage biocontrol applications in food production and processing. Curr. Issues Mol. Biol..

[21] Sharma G., Sharma S., Sharma P., Chandola D., Dang S., Gupta S., Gabrani R. (2016). *Escherichia coli* biofilm: Development and therapeutic strategies. J. Appl. Microbiol..

[22] Capita R., Riesco-Peláez F., Alonso-Hernando A., Alonso-Calleja C. (2014). Exposure of *Escherichia coli* ATCC 12806 to sublethal concentrations of food-grade biocides influences its ability to form biofilm, resistance to antimicrobials, and ultrastructure. Appl. Environ. Microbiol..

[23] Belay M., Sisay T., Wolde T. (2018). Bacteriophages and phage products: Applications in medicine and biotechnological industries, and general concerns. Sci. Res. Essays.

[24] Harada L.K., Silva E.C., Campos W.F., Del Fiol F.S., Vila M., Dąbrowska K., Krylov V.N., Balcão V.M. (2018). Biotechnological applications of bacteriophages: State of the art. Microbiol. Res..

[25] Park S., Nakai T. (2003). Bacteriophage control of *Pseudomonas plecoglossicida* infection in ayu. Dis. Aquat. Organ..

[26] Hawkins C., Harper D., Burch D., Anggard E., Soothill J. (2010). Topical treatment of *Pseudomonas aeruginosa* otitis of dogs with a bacteriophage mixture: A before/after clinical trial. Vet. Microbiol..

[27] Endersen L., O’Mahony J., Hill C., Ross R.P., McAuliffe O., Coffey A. (2014). Phage therapy in the food industry. Annu. Rev. Food Sci. Technol..

[28] Altamirano F., Barr J. (2019). Phage therapy in the postantibiotic era. Clin. Microbiol. Rev..

[29] Pires D.P., Melo L.D.R., Vilas Boas D., Sillankorva S., Azeredo J. (2017). Phage therapy as an alternative or complementary strategy to prevent and control biofilm-related infections. Curr. Opin. Microbiol..

[30] Ballén V., Cepas V., Ratia C., Gabasa Y., Soto S.M. (2022). Clinical *Escherichia coli:* From biofilm formation to new antibiofilm strategies. Microorganisms.

[31] Górski A., Jónczyk-Matysiak E., Lusiak-Szelachowska M., Miedzybrodzki R., Weber-Dabrowska B., Borysowski J. (2018). Phage therapy in allergic disorders?. Exp. Biol. Med..

[32] Domingo-Calap P., Delgado-Martínez J. (2018). Bacteriophages: Protagonists of a post-antibiotic era. Antibiotics.

[33] Pereira C., Costa P., Pinheiro L., Balcão V.M., Almeida A. (2021). Kiwifruit bacterial canker: An integrative view focused on biocontrol strategies. Planta.

[34] McLean S., Dunn L., Palombo E. (2013). Phage inhibition of *Escherichia coli* in ultrahigh-temperature-treated and raw milk. Foodborne Pathog. Dis..

[35] Tomat D., Mercanti D., Balagué C., Quiberoni A. (2013). Phage biocontrol of enteropathogenic and shiga toxin-producing *Escherichia coli* during milk fermentation. Lett. Appl. Microbiol..

[36] O’Flynn G., Ross R.P., Fitzgerald G.F., Coffey A. (2004). Evaluation of a cocktail of three bacteriophages for biocontrol of *Escherichia coli* O157:H7. Appl. Environ. Microbiol..

[37] Hudson J., Billington C., Wilson T., On S. (2013). Effect of phage and host concentration on the inactivation of *Escherichia coli* O157:H7 on cooked and raw beef. Food Sci. Technol. Int..

[38] Abuladze T., Li M., Menetrez M.Y., Dean T., Senecal A., Sulakvelidze A. (2008). Bacteriophages reduce experimental contamination of hard surfaces, tomato, spinach, broccoli, and ground beef by *Escherichia coli* O157:H7. Appl. Environ. Microbiol..

[39] Carter C.D., Parks A., Abuladze T., Li M., Woolston J., Magnone J., Senecal A., Kropinski A.M., Sulakvelidze A. (2012). Bacteriophage cocktail significantly reduces *Escherichia coli* O157: H7 contamination of lettuce and beef, but does not protect against recontamination. Bacteriophage.

[40] Ferguson S., Roberts C., Handy E., Sharma M. (2013). Lytic bacteriophages reduce *Escherichia coli* O157. Bacteriophage.

[41] Sharma M., Patel J.R., Conway W.S., Ferguson S., Sulakvelidze A. (2009). Effectiveness of bacteriophages in reducing *Escherichia coli* O157:H7 on fresh-cut cantaloupes and lettucet. J. Food Prot..

[42] Bach S., Mcallister T., Veira D., Gannon V., Holley R. (2003). Effect of bacteriophage DC22 on *Escherichia coli* O157:H7 in an artificial rumen system (Rusitec) and inoculated sheep. Anim. Res..

[43] Pereira C., Moreirinha C., Teles L., Rocha R.J.M., Calado R., Romalde J.L., Nunes M.L., Almeida A. (2017). Application of phage therapy during bivalve depuration improves *Escherichia coli* decontamination. Food Microbiol..

[44] Pereira C., Moreirinha C., Rocha R.J.M., Calado R., Romalde J.L., Nunes M.L., Almeida A. (2016). Application of bacteriophages during depuration reduces the load of *Salmonella* Typhimurium in cockles. Food Res. Int..

[45] Viazis S., Akhtar M., Feirtag J., Diez-gonzalez F. (2011). Reduction of *Escherichia coli* O157:H7 viability on hard surfaces by treatment with a bacteriophage mixture. Int. J. Food Microbiol..

[46] Patel J., Sharma M., Millner P., Calaway T., Singh M. (2011). Inactivation of *Escherichia coli* O157:H7 attached to spinach harvester blade using bacteriophage. Foodborne Pathog. Dis..

[47] Jaroni D., Litt P.K., Bule P., Rumbaugh K. (2023). Effectiveness of bacteriophages against biofilm-forming Shiga-toxigenic *Escherichia coli* in vitro and on food-contact surfaces. Foods.

[48] González-Gómez J.P., González-Torres B., Guerrero-Medina P.J., López-Cuevas O., Chaidez C., Avila-Novoa M.G., Gutiérrez-Lomelí M. (2021). Efficacy of novel bacteriophages against *Escherichia coli* biofilms on stainless steel. Antibiotics.

[49] Gill J.J., Hyman P. (2010). Phage choice, isolation, and preparation for phage therapy. Curr. Pharm. Biotechnol..

[50] Crémet L., Corvec S., Batard E., Auger M., Lopez I., Pagniez F., Dauvergne S., Caroff N. (2013). Comparison of three methods to study biofilm formation by clinical strains of *Escherichia coli*. Diagn. Microbiol. Infect. Dis..

[51] Naves P., Del Prado G., Huelves L., Gracia M., Ruiz V., Blanco J., Rodríguez-Cerrato V., Ponte M., Soriano F. (2008). Measurement of biofilm formation by clinical isolates of *Escherichia coli* is method-dependent. J. Appl. Microbiol..

[52] Pereira C., Moreirinha C., Lewicka M., Almeidab P., Clemente C., Romalde J.L., Nunes M.L., Almeida A. (2016). Characterization and in vitro evaluation of new bacteriophages for the biocontrol of *Escherichia coli*. Virus Res..

[53] Costa P., Pereira C., Gomes A., Almeida A. (2019). Efficiency of single phage suspensions and phage cocktail in the inactivation of *Escherichia coli* and *Salmonella* Typhimurium: An in vitro preliminary study. Microorganisms.

[54] Adams M.H. (1959). Bacteriophages.

[55] Sadekuzzaman M., Yang S., Mizan M.F.R., Ha S. (2017). Do Reduction of *Escherichia coli* O157:H7 in biofilms using bacteriophage BPECO 19. J. Food Sci..

[56] Mukane L., Racenis K., Rezevska D., Petersons A., Kroica J. (2022). Anti-biofilm effect of bacteriophages and antibiotics against uropathogenic *Escherichia coli*. Antibiotics.

[57] Park D.W., Park J.H. (2021). Characterization and food application of the novel lytic phage becp10: Specifically recognizes the o-polysaccharide of *Escherichia coli* O157:H7. Viruses.

[58] Mangieri N., Foschino R., Picozzi C. (2021). Application of bacteriophages on Shiga toxin-producing *Escherichia coli* (STEC) biofilm. Antibiotics.

[59] Coffey B., Anderson G., Filloux A., Ramos J. (2014). Biofilm formation in the 96-well microtiter plate. Pseudomonas Methods and Protocols. Methods in Molecular Biology.

[60] Zhu W., Ding Y., Huang C., Wang J., Wang J., Wang X. (2022). Genomic characterization of a novel bacteriophage STP55 revealed its prominent capacity in disrupting the dual-species biofilm formed by *Salmonella* Typhimurium and *Escherichia coli* O157:H7 strains. Arch. Microbiol..

[61] Park D.W., Park J.H. (2021). Characterization of a novel phage depolymerase specific to *Escherichia coli* O157:H7 and biofilm control on abiotic surfaces. J. Microbiol..

[62] Filippov A., Sergueev K.V., He Y., Huang X.Z., Gnade B.T., Mueller A.J., Fernandez-Prada C., Nikolich M.P. (2011). Bacteriophage-resistant mutants in *Yersinia pestis*: Identification of phage receptors and attenuation for mice. PLoS ONE.

[63] Tian F., Li J., Nazir A., Tong Y. (2021). Bacteriophage—A promising alternative measure for bacterial biofilm control. Infect. Drug Resist..

[64] Buck L.D., Paladino M.M., Nagashima K., Brezel E.R., Holtzman J.S., Urso S.J., Ryno L.M. (2021). Temperature-dependent influence of FliA overexpression on PHL628 *E. coli* biofilm growth and composition. Front. Cell. Infect. Microbiol..

[65] Uhlich G.A., Chen C.Y., Cottrell B.J., Nguyen L.H. (2014). Growth media and temperature effects on biofilm formation by serotype O157: H7 and non-O157 Shiga toxin-producing *Escherichia coli*. FEMS Microbiol. Lett..

[66] Rogers J., Dowsett A.B., Dennis P.J., Lee J.V., Keevil C.W. (1994). Influence of plumbing materials on biofilm formation and growth of *Legionella pneumophila* in potable water systems. Appl. Environ. Microbiol..

[67] Ruhal R., Kataria R. (2021). Biofilm patterns in gram-positive and gram-negative bacteria. Microbiol. Res..

[68] Faille C., Jullien C., Fontaine F., Bellon-Fontaine M.N., Slomianny C., Benezech T. (2002). Adhesion of *Bacillus* spores and *Escherichia coli* cells to inert surfaces: Role of surface hydrophobicity. Can. J. Microbiol..

[69] Pathirajah J.P., Balamurugan S., Arvaj L., Weiss J., Barbut S. (2022). Influence of different stainless steel finishes on biofilm formation by *Listeria monocytogenes*. J. Food Prot..

[70] Waldhans C., Hebel M., Herbert U., Spoelstra P., Barbut S., Kreyenschmidt J. (2023). Microbial investigation of cleanability of different plastic and metal surfaces used by the food industry. J. Food Sci. Technol..

[71] De-la-Pinta I., Cobos M., Ibarretxe J., Montoya E., Eraso E., Guraya T., Quindós G. (2019). Effect of biomaterials hydrophobicity and roughness on biofilm development. J. Mater. Sci. Mater. Med..

[72] Wang C., Hang H., Zhou S., Niu Y.D., Du H., Stanford K., McAllister T.A. (2020). Bacteriophage biocontrol of Shiga toxigenic *Escherichia coli* (STEC) O145 biofilms on stainless steel reduces the contamination of beef. Food Microbiol..

[73] Tabassum R., Shafique M., Khawaja K.A., Alvi I.A., Rehman Y., Sheik C.S., Abbas Z., ur Rehman S. (2018). Complete genome analysis of a *Siphoviridae* phage TSK1 showing biofilm removal potential against *Klebsiella pneumoniae*. Sci. Rep..

[74] Kozelskaya A.I., Verzunova K.N., Akimchenko I.O., Frueh J., Petrov V.I., Slepchenko G.B., Bakina O.V., Lerner M.I., Brizhan L.K., Davydov D.V. (2023). Antibacterial calcium phosphate coatings for biomedical applications fabricated via micro-arc oxidation. Biomimetics.

[75] Montso P.K., Mlambo V., Ateba C.N. (2021). Efficacy of novel phages for control of multi-drug resistant *Escherichia coli* O177 on artificially contaminated beef and their potential to disrupt biofilm formation. Food Microbiol..

[76] Chen L., Yuan S., Liu Q., Mai G., Yang J., Deng D., Zhang B., Liu C., Ma Y. (2018). In Vitro design and evaluation of phage cocktails against *Aeromonas salmonicida*. Front. Microbiol..

[77] Koskella B., Brockhurst M.A. (2014). Bacteria-phage coevolution as a driver of ecological and evolutionary processes in microbial communities. FEMS Microbiol. Rev..

[78] Duarte J., Pereira C., Costa P., Almeida A. (2021). Bacteriophages with potential to inactivate *Aeromonas hydrophila* in cockles: In Vitro and in vivo preliminary studies. Antibiotics.

[79] Capparelli R., Nocerino N., Iannaccone M., Ercolini D., Parlato M., Chiara M., Iannelli D. (2010). Bacteriophage therapy of *Salmonella enterica*: A fresh appraisal of bacteriophage therapy. J. Infect. Dis..

[80] Capparelli R., Nocerino N., Lanzetta R., Silipo A., Amoresano A., Giangrande C., Becker K., Blaiotta G., Evidente A., Cimmino A. (2010). Bacteriophage-resistant *Staphylococcus aureus* mutant confers broad immunity against staphylococcal infection in mice. PLoS ONE.

[81] León M., Kokkari C., García K., Castillo D., Katharios P., Bastías R. (2019). Diversification of *Vibrio anguillarum* driven by the bacteriophage CHOED. Front. Microbiol..

[82] Bohannan B.J.M., Travisano M., Lenski R.E. (1999). Epistatic interactions can lower the cost of resistance to multiple consumers. Evolution.

[83] Brockhurst M.a., Buckling A., Rainey P.B. (2005). The effect of a bacteriophage on diversification of the opportunistic bacterial pathogen, *Pseudomonas aeruginosa*. Proc. Biol. Sci..

[84] Lennon J.T., Khatana S.A.M., Marston M.F., Martiny J.B.H. (2007). Is there a cost of virus resistance in marine cyanobacteria?. ISME J..

[85] Quance M.A., Travisano M. (2009). Effects of temperature on the fitness cost of resistance to bacteriophage T4 in *Escherichia coli*. Evolution.

[86] Bhandare S., Goodridge L., Harper D., Abedon S., Burrowes B., McConville M. (2021). Bacteriophages as bio-sanitizers in food production and healthcare settings. Bacteriophages.

[87] Costa M.J., Pastrana L.M., Teixeira J.A., Sillankorva S.M., Cerqueira M.A. (2023). Bacteriophage delivery systems for food applications: Opportunities and perspectives. Viruses.

